# Surgical management and oncological outcome of non-squamous cell carcinoma of the larynx: a bicentric study

**DOI:** 10.1007/s00405-021-07076-x

**Published:** 2021-09-24

**Authors:** Andrea Iandelli, Francesco Missale, Andrea Laborai, Marta Filauro, Filippo Marchi, Francesca Del Bon, Pietro Perotti, Giampiero Parrinello, Cesare Piazza, Giorgio Peretti

**Affiliations:** 1 IRCCS Ospedale Policlinico San Martino, Genoa, Italy; 2 Department of Surgical Sciences and Integrated Diagnostics (DISC), University of Genoa, Genoa, Italy; 3 Department of Molecular and Translational Medicine, University of Brescia, Viale Europa 11, 25123 Brescia, Italy; 4 Unit of Otorhinolaryngology, Guglielmo da Saliceto Hospital, Piacenza, Italy; 5 Unit of Otorhinolaryngology, Head and Neck Surgery, ASST Spedali Civili di Brescia, University of Brescia, Brescia, Italy; 6 Unit of Otorhinolaryngology-Head and Neck Surgery, “S. Chiara” Hospital, Azienda Provinciale per I Servizi Sanitari (APSS), Trento, Italy

**Keywords:** Laryngeal neoplasms, Rare tumors, Laryngectomy, Recurrence, Survival

## Abstract

**Purpose:**

Non-squamous cell carcinoma (non-SCC) accounts for about 5% of laryngeal malignancies. Survival data are limited, and consensus on management principles is lacking. The present study reviews our experience in the surgical treatment of non-metastatic non-SCC of the larynx and compares oncological and functional outcomes in a cohort of patients affected by traditional SCC.

**Methods:**

We collected data on 592 patients affected by laryngeal neoplasms. Univariate and multivariable survival analyses were performed using Cox proportional-hazards models; survival estimates were reported by hazard ratios (HR) with 95% confidence intervals (CI), and survival curves were established with the Kaplan–Meier method.

**Results:**

We identified 326 patients affected by untreated SCC, while 21 had non-SCC histotypes. The non-SCC cohort was composed of 5 soft tissue sarcomas, 8 chondrosarcomas, 2 adenoid cystic carcinomas, 2 neuroendocrine carcinomas, 2 solitary fibrous tumors, 1 Kaposi’s sarcoma, and 1 malignant peripheral nerve sheath tumor. Overall survival and disease-specific survival were not significantly different according to histology (*p* = 0.6 and *p* = 0.349, respectively). The non-SCC group showed an increased risk of recurrence (HR 5.87; CI_95_ 2.15–16.06; *p* < 0.001). Nonetheless, no significant difference (*p* = 0.31) was found at multivariable analysis between the two groups in total laryngectomy-free survival with an organ preservation rate over 5 years of 81% for the non-SCC histologies.

**Conclusion:**

Non-SCC is a broad spectrum pathology, but generalized laryngeal surgical management principles are still feasible and it is possible to identify patients amenable to conservative surgical treatment without affecting survival.

**Supplementary Information:**

The online version contains supplementary material available at 10.1007/s00405-021-07076-x.

## Introduction

More than 95% of all malignant laryngeal neoplasms are represented by squamous cell carcinoma (SCC) [1]. In contrast, about 5% of the remaining are composed of other histotypes originating from minor salivary glands, bone, cartilage, muscle, fatty tissue, neuronal, and connective tissue. These are cell types seen throughout the aerodigestive tract and, specifically, within the larynx. The entire group of these tumors is considered as rare non-SCC tumors [2]. The usual submucosal growth pattern and supraglottic or subglottic location frequently cause a delay in diagnosis, which might happen at an intermediate-advanced local stage [3]. Due to the paucity of cases, outcomes associated with non-SCC laryngeal cancers are not well characterized.

Regarding the laryngeal site, facing a patient affected by non-SCC cancer raises several doubts. The site-specific TNM system is often not applicable (e.g., soft tissue tumors), and both radiosensitivity and response to systemic therapy are unpredictable [2]. Their frequent unconventional clinical presentation creates initial uncertainty among otolaryngologists attempting to make a diagnosis and develop a treatment plan [4]. Moreover, survival data are limited, and a consensus on management principles has yet to be fully elucidated.

The present study reviews our experience in the surgical management of non-metastatic non-SCC of the larynx, analyzing both oncological and functional outcomes expressed as total laryngectomy-free survival (TLFS). We used a control group of patients affected by conventional SCC as a benchmark to compare the results in the non-SCC cohort and whether non-SCC might represent an independent factor affecting the aforementioned end points.

## Materials and methods

### Patients

We retrospectively evaluated data on 592 patients affected by laryngeal neoplasms treated at two Italian institutions, between 2012 and 2017 (the Unit of Otorhinolaryngology-Head and Neck Surgery of the University of Genoa, and the Unit of Otorhinolaryngology-Head and Neck Surgery of the University of Brescia, Italy). Inclusion criteria were clinical evidence of previous untreated epithelial or non-epithelial laryngeal neoplasm, at least 1 year of follow-up or an earlier date of recurrence or death. Patients affected by recurrent tumors (previous surgery or radiotherapy), laryngeal lymphomas, secondary laryngeal involvement by other malignancies, or patients with missing data were excluded.

### Diagnostic work-up and treatment policy

Clinical endoscopic evaluation was performed preoperatively by a flexible video-endoscope or intraoperatively by rigid telescopes under white light (WL) and narrow band imaging (NBI, Olympus Medical System Corporation, Tokyo, Japan); the superficial boundaries of the lesion were one of the critical targets of the evaluation. In cases with a suspicious deep extension to the mucosal layer, the staging was completed by neck computed tomography (CT) or magnetic resonance imaging (MRI) performed by dedicated radiologists. Tumors were classified and treated according to the VII Edition of the Union International Cancer Control–American Joint Committee on Cancer (UICC-AJCC) TNM staging system for the laryngeal site [5], as enrolled between 2012 and 2017.

All patients underwent complete surgical excision of the primary tumor, with or without neck dissection, following NCCN guidelines [6]. Re-excision was performed in case of a positive margin(s). Patients with persistent tumor after re-excision, perineural invasion, angioembolization, multiple positive lymph nodes, pT4a stage, poor differentiation, or extracapsular spread underwent adjuvant radiotherapy (RT) or chemoradiotherapy (CRT). Considering follow-up policy, the entire cohort of patients received endoscopic evaluation every 2 months during the first year, every 3–4 months during the second and third year, every 6 months in the fourth and fifth year, and then annually [7]. CT or MRI was performed every 6 months in the first year and then annually for at least 3 years.

### Statistical analysis

Summary statistics were performed reporting absolute and relative frequencies. Qualitative variables were compared between groups by chi-square test or Fisher’s exact test. Survival analysis was performed considering as outcomes; overall survival (OS) is defined as the time between the date of the treatment and the date of death; disease-specific survival (DSS) is defined as the time between the date of the treatment and the date of death due to disease progression; disease-free survival (DFS) is defined as the time between the date of the treatment and the date of recurrence. TLFS was analyzed for patients submitted to conservative surgery, defined as the time between the treatment and the total laryngectomy date. In the absence of any event, survivals were censored at the last follow-up visit. Univariate and multivariable survival analyses were performed using Cox proportional-hazards models; assumptions for the models were verified for all analyses performed. Backward multivariable model building was carried out by measuring Akaike’s information criteria (AIC) and applying the likelihood ratio test for comparisons of nested models. Survival estimates were reported using hazard ratios (HR) with 95% confidence intervals (CI) and drawing adjusted survival curves from the multivariable survival models. Univariable survival curves were plotted with the Kaplan–Meier method. In all analyses, a significance level of 5% was used. R (version 3.6.3) was used for statistical analysis [8].

## Results

### Demographic features of the study cohort

Of the 592 laryngeal cancer patients recruited, 360 met inclusion criteria. Of these, 10 patients were excluded for missing data. Among 350 patients available for the analysis, 24 were affected by rare neoplasms, 3 benign and 21 malignant, while 326 were affected by invasive SCC. To obtain an equivalent evaluation with SCC, only the rare malignant neoplasms (non-SCC), to which we will refer to as non-SCC, were considered for further analysis (Table 1), and thus a total of 347 patients were suitable for the study (Table 2). The entire group was composed of 299 males and 48 females. The demographic and clinical data of the cohort are presented in Table 2.

**Table 1:** Demographic and clinical details of the non-SCC group

Sex	Age	Histology	TNM	Treatment	Recurrence treatment	Last follow-up (m)	Status
M	60	Chondrosarcoma	cT2 N0 M0	CTRA	TLM (5 m)	6	AWD
M	51	Chondrosarcoma	cT4a N0 M0	CTRA	CTRA (77 m); TL (132 m)	200	NED
F	41	Solitary fibrous tumor	cT2 N0 M0	PPL	–	58	NED
F	68	Sinovial sarcoma	cT2 N0 M0	PPL	–	18	NED
M	63	Rhabdomyosarcoma	cT2 N0 M0	TLM	–	42	NED
M	67	Chondrosarcoma	cT3 N0 M0	TLM	TL (4 m)	89	NED
M	66	Solitary fibrous tumor	cT2 N0 M0	TLM	PPL (6 m)	55	NED
M	74	Liposarcoma	cT2 N0 M0	TLM	TLM (12 m)	29	DOC
F	78	Kaposi’s sarcoma	cT2 N0 M0	TLM	–	40	NED
M	54	Malignant peripheral nerve sheat tumor	cT4a N0 M0	PPL	–	20	NED
M	67	Pleomorphic sarcoma	cT1 N0 M0	TLM	–	19	NED
F	63	Adenoid cystic carcinoma	cT3 N0 M0	TLM	TL (1 m)	57	NED
F	65	Chondrosarcoma	cT3 N0 M0	Laryngofissure	-	7	NED
M	67	Adenoid cystic carcinoma	cT4a N0 M0	OPHL	TL (28 m)	29	NED
M	72	Small cell neuroendocrine carcinoma	cT1a N0 M0	TLM	–	29	NED
M	64	Liposarcoma	cT2 N0 M0	TLM	PPL (50 m)	92	AWD
F	66	Small cell neuroendocrine carcinoma	cT4a N0 M0	RT-CHT	CHT (5 m)	77	DOD
M	67	Chondrosarcoma	cT1 N0 M0	TLM	–	39	NED
M	71	Chondrosarcoma	cT1 N0 M0	TLM	PPL (7 m)	61	NED
F	66	Chondrosarcoma	cT2 N0 M0	TLM	TLM (2 m)	14	AWD
F	74	Chondrosarcoma	cT3 N0 M0	TLM	–	1	NED

Legend: *M* male; *F* female; *TLM* transoral laser microsurgery; *CTRA* crico-tracheal resection and anastomosis; *OPHL* open partial horizontal laryngectomy; *TL* total laryngectomy; *PPL* partial pharyngo-laryngectomy; *RT* radiotherapy; *CHT* chemotherapy; *m* months; *AWD* alive with disease; *NED* not evidence of disease; *DOC* dead of other causes; *DOD* dead of disease

**Table 2:** Clinical features and association analysis between the conventional SCC cohort and the non-SCC cohort

Variables		Total		SCC		Non-SCC		*P* value
		N	%	N	%	N	%	
Sex	Male	299	86.2	286	87.7	13	61.9	0.004
	Female	48	13.8	40	12.3	8	38.1	
Alcohol	No	254	73.2	234	71.8	20	95.2	0.02
	Yes	93	26.8	92	28.2	1	4.8	
Smoke	No	52	15.0	43	13.2	9	42.9	0.001
	Yes/ex	295	85.0	283	86.8	12	57.1	
Site	Supraglottis	69	19.9	58	17.8	11	52.4	< 0.001
	Glottis	251	72.3	248	76.1	3	14.3	
	Subglottis	7	2	0	0	7	33.3	
	Transglottis	20	5.77	20	6.1	0	0	
Fixation	No	296	85.3	281	86.2	15	71.43	0.1
	Yes	51	14.7	45	13.8	6	28.6	
Stage	I	166	47.8	158	48.5	8	38.1	0.66
	II	58	16.7	53	16.3	5	23.8	
	III	69	19.9	65	20.0	4	19.1	
	IV	54	15.6	50	15.3	4	19.1	
Grade	G1	58	16.7	49	15.0	9	42.9	< 0.001
	G2	237	68.3	232	71.2	5	23.8	
	G3	20	11.5	35	10.7	5	23.8	
	Missing	12	3.5	10	3.1	2	9.5	
Treatment	TLM	266	76.7	253	77.6	13	61.9	< 0.001
	OPHL	26	7.5	25	7.7	1	4.8	
	CTRA	2	0.6	0	0.0	2	9.5	
	TL	48	13.8	48	14.7	0	0.0	
	Other	5	1.4	0	0.0	5	23.8	

*P* values estimated by Fisher’s exact test

Legend: *SCC* squamous cell carcinoma; *TLM* transoral laser microsurgery; *OPHL* open partial horizontal laryngectomy; *CTRA* crico-tracheal resection and anastomosis; *TL* total laryngectomy

### Characteristics of the non-SCC group

**Fig. 1:**
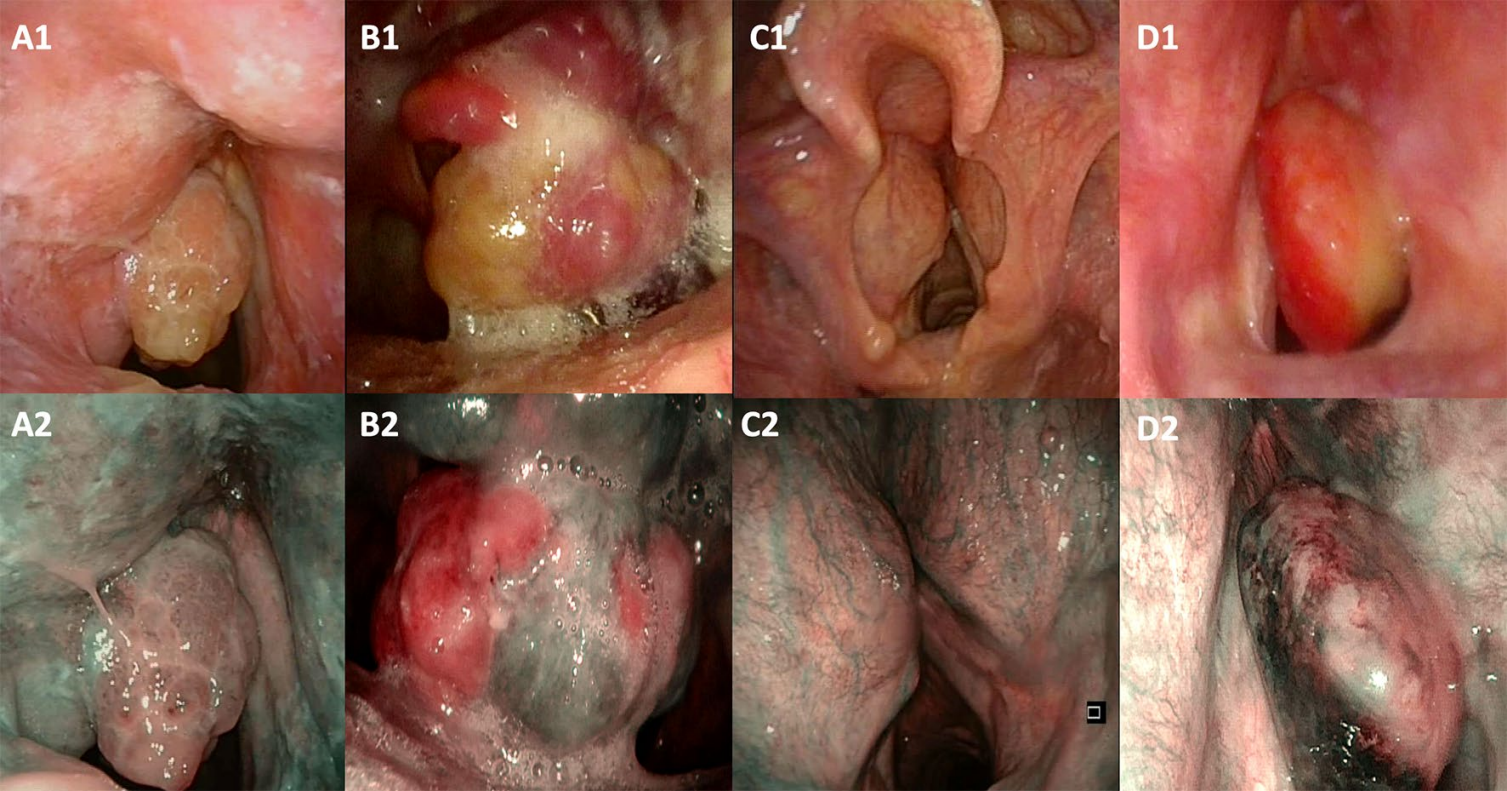
Clinical endoscopic pictures of cases of non-SCC. **A1** Fusiform cell rhabdomyosarcoma white light appearance; **A2** fusiform cell rhabdomyosarcoma narrow band imaging appearance; **B1** Kaposi sarcoma white light appearance; **B2** Kaposi sarcoma narrow band imaging appearance; **C1** chondroma white light appearance; **C2** chondroma narrow band imaging appearance; **D1** non-small cell neuroendocrine carcinoma white light appearance; **D2** non-small cell neuroendocrine carcinoma narrow band imaging appearance

The non-SCC cohort was composed of 5 soft tissue sarcomas, 8 chondrosarcomas, 2 adenoid cystic carcinomas, 2 neuroendocrine carcinomas, 2 solitary fibrous tumors, 1 Kaposi’s sarcoma, and 1 malignant peripheral nerve sheath tumor; representative pictures of endoscopic appearance in white light and with NBI are depicted in Fig. 1. A time-to-event chart of the non-SCC cohort is reported in Supplementary Figure S1. Transoral laser microsurgery (TLM) with the support of cold instruments when needed was performed in 13 patients. Two patients underwent crico-tracheal resection and anastomosis (CTRA) due to the substantial subglottic extension that was not manageable with a transoral approach alone. In one case, in which the laryngeal exposition was suboptimal, a laryngofissure operation was necessary. Three partial pharyngolaryngectomies (PPL) were performed as simultaneous lateral oro-hypopharyngeal wall involvement was present that was not amenable to endoscopic resection; in all these cases, the resulting defect did not require free flap reconstruction and a water-tight suture of pharyngeal mucosa was possible by primary closure. One open partial laryngectomy (OPHL) was performed because of the concomitant presence of poor laryngeal exposition and the advanced local stage of the disease. One patient refused surgical treatment, and after an endoscopic debulking for airway patency restoring, she was submitted to chemoradiotherapy (CCRT).

Considering the survival end points, the 2 year and 5 year OS estimates, respectively, were 100% (CI_95%_ 100–100%) and 93% (CI_95%_ 80–100%), DSS 100% (CI_95%_ 100–100%) and 100% (CI_95%_ 100–100%), DFS 60% (CI_95%_ 41–86%) and 34% (CI_95%_ 13–86%), and TLFS 90% (CI_95%_ 78–100%) and 81% (CI_95%_ 63–100%).

### Comparison of groups

Female patients were significantly more represented in the non-SCC group compared to the SCC group (38.1% vs 12.3, *p* = 0.004). The mean age at the time of treatment was 68 years with no significant difference between groups. Tobacco consumption was less predominant among patients affected by non-SCC (57.1 vs 86.8%, *p* = 0.001) than SCC, while none of the subjects within this group declared alcohol abuse. Comparison between the conventional SCC group showed significant differences in terms of sites involvement (*p* < 0.001), as the supraglottic site was significantly more frequent in non-SCC compared to SCC (52.4 vs 17.8%, respectively); moreover, 33.3% of non-SCC patients presented subglottic involvement, while none of the patients affected by SCC showed subglottic involvement alone. Only 14.1% of the non-SCC group demonstrated glottic involvement alone, while 76.1% of SCC were located at this level. None of the patients in the non-SCC group showed transglottic extension. The surgical treatment performed was also heterogeneous among the two groups (*p* < 0.001). Besides transoral laser microsurgery (TLM), the most common treatment modality in both cohorts (77.6% for SCC and 61.9% for non-SCC), 13.8% of SCC was treated by upfront total laryngectomy (TL); in contrast, none of the non-SCC patients were treated by TL as first-line treatment. CTRA (*n* = 2) or other surgical procedures (*n* = 5); specifically, laryngofissure and PPL were adopted only in the non-SCC group. By comparing histological grading, we observed a significantly different distribution (*p* < 0.001) between the two groups; SCC patients were mainly affected by moderately differentiated neoplasms (68.3%). At the same time, the non-SCC cohort showed considerable heterogeneity: non-SCC were classified as well differentiated (G1) in 38.1%, moderately differentiated in 23.8%, and poorly differentiated (G3) in 23.8%. Among other clinical features, such as laryngeal fixation and stage, no significant differences were observed; full details of the comparisons are reported in Table 2.

### Overall survival analysis

**Fig. 2:**
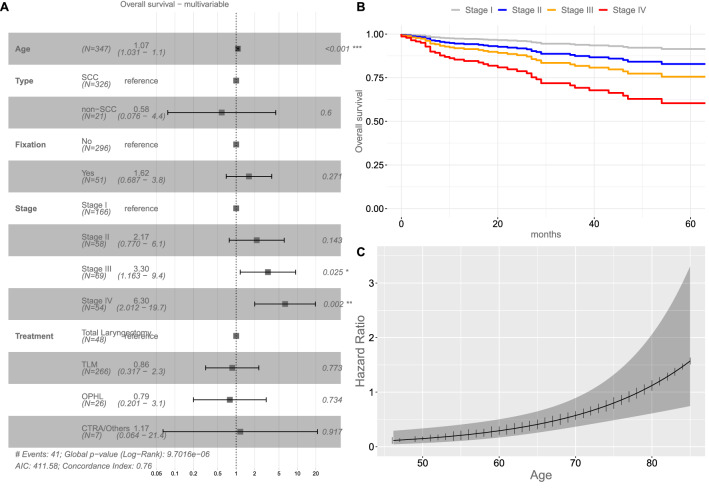
**A** Forest plot of multivariable overall survival (OS) analysis; survival curves derived from the multivariable OS model showing the marginal effect of the Stage **B** and of the age **C** adjusted for the effects of other covariates. Legend: *SCC* squamous cell carcinoma; *TLM* transoral laser microsurgery; *OPHL* open partial horizontal laryngectomy; *CTRA* crico-tracheal resection and anastomosis; *TL* total laryngectomy

Considering OS as the primary end point, at univariate analysis higher age (H.R. 1.06, CI_95%_ 1.02–1.09, *p* = 0.001), presence of arytenoid fixation (H.R. 3.14, CI_95%_ 1.59–6.23, *p* = 0.001), different stage (*p* < 0.05), and TLM treatment (H.R. 0.29, CI_95%_ 0.14–0.58, *p* = 0.001) were associated with different survival outcomes (Table 3, Supplementary Figure S2). Age and stage were also independent predictors in the multivariable model proposed, and TLM treatment was not a protective factor at multivariable analysis after adjusting for stage and age. Of note, at univariate or multivariable analysis, the non-SCC tumors were not associated with different OS compared to SCC (Table 3 and Fig. 2).

**Table 3:** Univariate OS and DSS analysis

Variables	Overall survival (OS)			Disease-specific survival (DSS)		
	HR	95% CI	*P*	HR	95% CI	*P*
Age	1.06	1.02–1.09	0.001	1.02	0.97–1.08	0.363
Sex (female vs male)	1.13	0.47–2.70	0.78	2.04	0.66–6.32	0.219
Smoke (yes vs no)	2.2	0.68–7.16	0.19	2.89	0.38–21.93	0.304
Alcohol (yes vs no)	0.81	0.38–1.71	0.58	1.21	0.42–3.48	0.728
Type (non-SCC vs SCC)	0.56	0.13–2.41	0.44	0.54	0.07–4.42	0.568
Supraglottic	Reference	–	–	Reference	–	–
Glottic	0.8	0.38–1.67	0.551	0.99	0.31–3.19	0.99
Subglottic	0	0	1	0	0	1
Transglottic	1.61	0.50–5.16	0.427	1.12	0.12–10.25	0.92
Fixation (yes vs no)	3.14	1.59–6.23	0.001	4.38	1.58–12.16	0.005
Stage I	Reference	–	–	Reference	–	–
Stage II	2.68	0.97–7.40	0.057	1.12	0.1–12.33	0.929
Stage III	3.14	1.20–8.26	0.02	3.29	0.55–19.68	0.193
Stage IV	6.09	2.45–15.15	< 0.001	14.49	3.16–66.53	0.001
G1	0.43	0.13–1-43	0.17	0.00	0 -	1
G2	Reference	–	–	Reference	–	–
G3	1.69	0.77–3.71	0.19	1.88	0.60–5.91	0.28
TLM	0.29	0.14–0.58	0.001	0.1	0.03–0.29	< 0.001
OPHL	0.41	0.11–1.44	0.163	0.2	0.02–1.57	0.125
CTRA/others	0.33	0.04–2.75	0.31	0.3	0.03–3.18	0.32
TL	Reference	–	–	Reference	–	–

Legend: *SCC* squamous cell carcinoma; *TLM* transoral laser microsurgery; *OPHL* open partial horizontal laryngectomy; *CTRA* crico-tracheal resection and anastomosis; *TL* total laryngectomy

### Disease-specific survival analysis

**Fig. 3:**
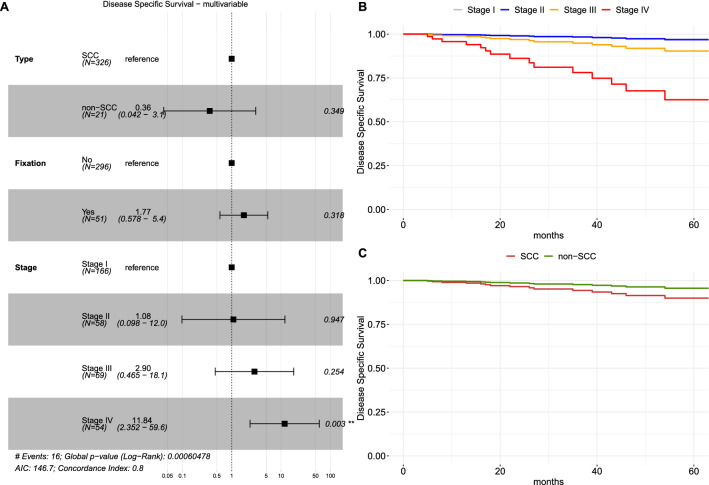
**A** Forest plot of multivariable disease-specific survival analysis (DSS); survival curves derived from the multivariable DSS model showing the marginal effect of the stage (**B**) and of the histological type (**C**), adjusted for the effects of other covariates. Legend: *SCC* squamous cell carcinoma

Considering DSS, no significant differences between the non-SCC group and SCC were observed at univariate and multivariable analysis (Table 3, Fig. 3, Supplementary Figure S3). The presence of arytenoid fixation (H.R. 4.38, CI_95%_ 1.58–12.16, *p* = 0.005) and Stage IV (H.R. 14.49, CI_95%_ 3.16–66.53, *p* = 0.001) were significantly associated with DSS, and Stage IV was the only independent risk factor in the multivariable DSS model (*p* = 0.003), as reported in Fig. 3.

### Disease-free survival analysis

**Fig. 4:**
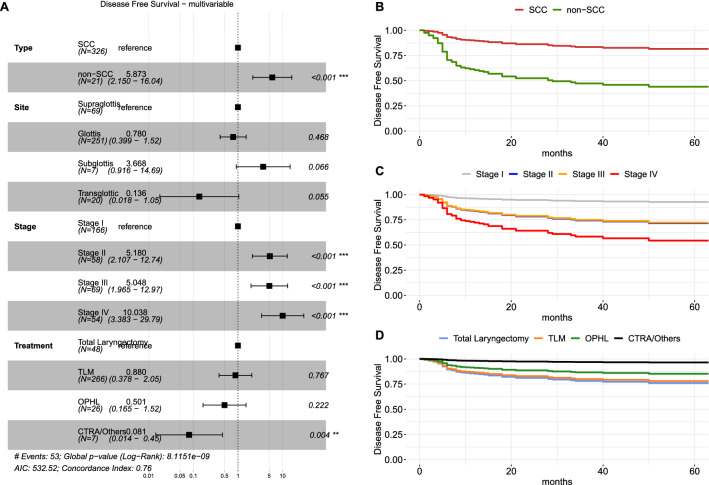
**A** Forest plot of multivariable disease-free survival analysis (DFS); survival curves derived from the multivariable DFS model showing the marginal effect of the histological type (**B**), stage (**C**) and treatment (**D**), adjusted for the effects of other covariates. Legend: *SCC* squamous cell carcinoma; *TLM* transoral laser microsurgery; *OPHL* open partial horizontal laryngectomy; *CTRA* crico-tracheal resection and anastomosis; *TL* total laryngectomy

Analyzing DFS, several covariates were associated with a higher risk of recurrence, such as the non-SCC group (H.R. 4.46, CI_95%_ 2.29–8.69, *p* < 0.001), presence of arytenoid fixation (H.R. 2.19, CI_95%_ 1.15–4.19, *p* = 0.018) and higher overall stage (*p* < 0.05) (Table 4, Supplementary Figure S4). At multivariable analysis, higher stage (*p* < 0.05) and non-SCC (H.R. 5.65, CI_95%_ 2.04–15.68, *p* < 0.001) were confirmed to be independent risk factors of worse DFS (Fig. 4).

**Table 4:** Univariate DFS and TLFS analysis

Variables	Disease-free survival (DFS)			Total laryngectomy-free survival (TLFS)		
	HR	95% CI	*P*	HR	95% CI	*P*
Age	0.99	0.97–1.02	0.702	0.96	0.91–1	0.073
Sex (female vs male)	1.67	0.84–3.33	0.146	1.27	0.28–5.8	0.76
Smoke (yes vs no)	0.55	0.29–1.05	0.071	0.93	0.2–4.24	0.92
Alcohol (yes vs no)	0.79	0.41–1.50	0.463	0.6	0.13–2.75	0.51
Type (non-SCC vs SCC)	4.46	2.29–8.69	< 0.001	4.4	1.19–16.26	0.026
Supraglottic	Reference	–	–	Reference	–	–
Glottic	0.31	0.18–0.56	< 0.001	0.47	0.12–1.87	0.28
Subglottic	3.13	1.16–8.45	0.024	6.61	1.1–39.78	0.039
Transglottic	0.15	0.02–1.10	0.061	1.71	0.18–16.45	0.64
Fixation (yes vs no)	2.19	1.15–4.19	0.018	4.07	0.88–18.76	0.07
Stage I	Reference	–	–	Reference	–	–
Stage II	4.04	1.73–9.46	0.001	8.1	0.84–77.87	0.07
Stage III	3.49	1.49–8.18	0.004	11.72	1.31–104.94	0.028
Stage IV	7.28	3.26–16.27	< 0.001	39.76	4.44–355.89	0.001
G1	1.02	0.49–2.12	0.95	0.91	0.19–4.30	0.91
G2	Reference	–	–	1	1	
G3	1.19	0.53–2.67	0.67	2.08	0.44–9.81	0.35
TLM	0.43	0.22–0.84	0.013	Reference	–	–
OPHL	0.72	0.25–2.05	0.543	5.49	1.65–18.25	0.006
CTRA/Others	1.37	0.38–5.01	0.63	< 0.01	< 0.01–	1
TL	Reference	–	–			

Legend: *SCC* squamous cell carcinoma; *TLM* transoral laser microsurgery; *OPHL* open partial horizontal laryngectomy; *CTRA* crico-tracheal resection and anastomosis; *TL* total laryngectomy

### Total Laryngectomy-free survival analysis

**Fig. 5:**
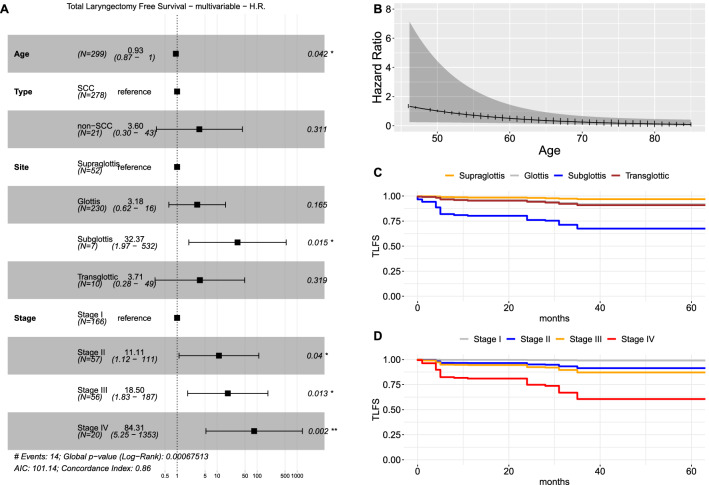
**A** Forest plot of multivariable total laryngectomy-free survival analysis (TLFS); survival curves derived from the multivariable TLFS model showing the marginal effect of the age (**B**), site (**C**) and stage (**D**), adjusted for the effects of other covariates. Legend: *SCC* squamous cell carcinoma

Among the 251 patients (83.9%) submitted to conservative laryngeal surgery, TLFS was also evaluated as a secondary outcome. At univariate analysis, non-SCC group (H.R. 4.4, CI_95%_ 1.19–16.26, *p* = 0.026), OPHL surgery (H.R. 5.49, CI_95%_ 1.65–18.25, *p* < 0.001), and higher stage (*p* < 0.05) were associated with a higher risk of TL (Table 4, Supplementary Figure S5). At multivariable analysis, stratified for treatment, advanced Stage (*p* < 0.05), subglottic site (H.R. 32.37, CI_95%_ 1.97–532, *p* = 0.015), and younger age (H.R. 0.93, CI_95%_ 0.87–0.99, *p* = 0.042) were significantly associated with a higher risk of TL (Fig. 5).

## Discussion

Due to their rarity, laryngeal non-SCCs present a significant challenge to clinicians because no definitive guidelines exist to advise a uniform treatment strategy. In this study, we aimed to characterize laryngeal non-SCCs based on clinical and pathological features and evaluate outcomes of surgical management in the group as a whole to better understand the impact in terms of survival and organ preservation of neoplasms other than SCC. Consequently, we compared the non-SCC cohort with traditional SCC to define the possible alternative strategy to embrace.

In agreement with other authors [9, 10], we applied the staging system for conventional laryngeal SCC since a histology-driven staging system is lacking. Moreover, this classification is the standard format for stratifying patients with laryngeal malignancies and is designed to establish treatment and prognosis.

According to the recent literature, the majority of publications are case reports or small case series [3, 4, 11–17], including both malignant and benign neoplasms [12] and even non-neoplastic lesions [3]. Nevertheless, cohort studies and cross-sectional population analysis [9, 18] considered only OS as survival end point. The population-based studies from National cancer Database are based on data that are over a decade old and lack detailed information on the pathological features and treatment modalities. Furthermore, only two studies compared non-SCC outcomes with SCC [9, 10]. To the best of our knowledge, our study is the first to compare several oncological outcomes (OS, DSS, and DFS) and a functional one, such as the TLFS, between SCC and non-SCC. Unlike past studies [3, 9, 10, 12, 13], we did not include laryngeal lymphoma or metastatic location to the larynx in the non-SCC group, whose treatment is almost exclusively accomplished through chemo/radiotherapy, reserving a marginal role for surgery [19–22].

In our cohort, women were more represented in the non-SCC group compared to the SCC (38.1 vs 12.3%, *p* = 0.004); this can be explained by the absence of known associations between non-SCC, except for neuroendocrine carcinoma, [23], and the consumption of alcohol and tobacco as risk factors, which are more common habits in males in Western countries [24, 25]. We found a significant difference (*p* < 0.001) among the various laryngeal subsites involved by SCC and non-SCC. The supraglottic and subglottic regions were strongly associated with non-SCC compared to SCC; our cohort was mainly represented by hard and soft tissue sarcomas, histotypes that arise more commonly in these subsites [10, 17]. Moreover, the subglottic involvement by SCC is an infrequent event with a range of 1–1.6% worldwide [26, 27]. We observed a different distribution of grading between the two groups. This finding is the mirror of the histological heterogeneity of which the non-SCC cohort is composed. In fact, this group included traditionally aggressive neoplasms, like small cell neuroendocrine carcinoma and pleomorphic sarcoma [28, 29], and histotypes with a more indolent biological behavior, such as chondrosarcoma, liposarcoma, and solitary fibrous tumor [30, 31]. The SCC cohort, conversely, was mainly composed of moderately differentiated carcinomas. None of the patients affected by non-SCC underwent an upfront laryngectomy: nine patients were treated by TLM, one patient with OPHL, 2 CTRA, 3 PPL, and one laryngofissure; only one patient affected by a locally advanced neuroendocrine carcinoma refused surgical treatment and was addressed to concomitant chemo/radiotherapy. Although 38.2% of the non-SCC patients were affected by stage III-IV disease, we pursued a conservative approach, guided by the nature of the neoplasm, and not performing any upfront laryngectomy in this group. The intermediate/advanced lesions were composed mainly of histological types in which surgery is the first treatment of choice [32] or whose indolent biological behavior does not justify a more aggressive treatment [33–35]. Indeed, none of the patients affected by locally advanced disease in the non-SCC group had concomitant regional involvement or developed regional or distant recurrence during follow-up. Other authors have shown that hard and soft tissue sarcomas in the head and neck region infrequently involve the neck lymph nodes [36–38]. On the other hand, the more aggressive histological types, i.e., adenoid cystic carcinoma, pleomorphic rhabdomyosarcomas, or neuroendocrine tumors, when presented at an early stage, are still manageable with TLM [15, 23]. Furthermore, soft tissue sarcomas show a better prognosis when they arise in the larynx compared to other locations in the body [39, 40].

**Fig. 6:**
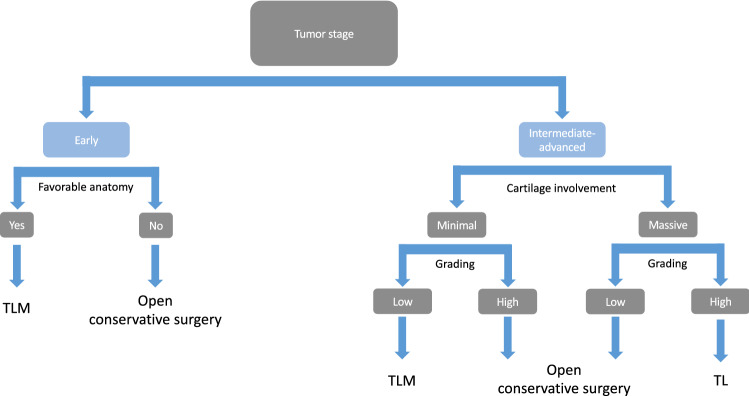
Algorithm of the main decision-making process adopted for the choice of the surgical approach to be adopted

Interestingly, the tumor type (SCC vs. non-SCC) did not appear to impact OS and DSS in multivariate analysis, whereas the overall stage represented the most ubiquitous risk factor affecting every domain evaluated. Our results are similar to those reported by Lin et al. [10]. These authors conducted a cross-sectional population analysis using data obtained by a Surveillance, Epidemiology, and End Results database, having as primary end point the differences in outcome in terms of OS. The authors demonstrated that tumor histopathology did not constitute an independent risk factor for OS in multivariable analysis. At the same time, both T-stage and N-stage of non-SCC independently predicted survival time of patients with supraglottic primaries, whereas T-stage alone was an independent predictor of survival for patients with glottic primaries. Nevertheless, Chen et al., who published the only other study present in the literature, performed a comparative analysis between SCC and non-SCC, finding a significant difference in OS between non-SCC and SCC with longer OS in the latter. The discordance between our results, in terms of OS, and those by Chen, might be a by-product of the differences in the composition of the non-SCC group, which, compared to our cohort, comprised a higher rate of neuroendocrine tumor (24.5% of the entire group) and only one case of hard tissue sarcoma. The variable non-SCC showed, at multivariable analysis, to represent an independent risk factor impacting DFS (HR 5.873; *p* < 0.001). We may surmise that the higher recurrence risk observed in non-SCCs could result from the conservative treatment choice rather than a sign of local tumor aggressiveness. During the decision-making process, we preferred remodeling surgery in the first instance, either endoscopic or open, as summarized in Fig. 6. This approach, already described by Damiani et al. [33] and adopted by other authors [41] in the management of chondrosarcoma, was demonstrated to be a feasible procedure even in liposarcoma [30] and in selected patients affected by salivary gland malignancies despite their detrimental biological behavior [32, 42–44]. The treatment aims to restore airway patency and reduce symptoms such as dyspnea and dysphagia rather than obtain wide surgical margins through extensive, mutilating surgery, especially when dealing with low-grade, slow-growing tumors. According to oxford dictionary: “an algorithm is a process or set of rules to be followed in calculations or other problem-solving operations, especially by a computer”. The more comprehensive the algorithm is, the more clinically relevant it becomes but more complex to construct. Therefore, a joint effort by experts taking literature into account, not the literature alone with all its limitations, should be used to construct an algorithm that dictates human dignity and quality of life. We encourage the readers to take the one we propose as a general principle of the decision-making process. Simplifying the approach could lead us to under-/overtreat with each decision possibly impacting patient’s quality of life, medical expenditures, etc., differently.

Despite the higher recurrence rate for the non-SCC group (H.R. 5.65, 5 year DFS estimate 34%), the good results in terms of 5 year OS (93%) and 5 year TLFS (81%) confirm that organ preservation treatment, faced with recurrent disease, is in most of cases feasible and conservative treatment as the first choice does not jeopardize long-term survival. The main limit of our study is the retrospective setting and its limited sample size, anyway derived from two institutions that share the diagnostic and therapeutic decision-making process for the management of laryngeal rare tumors. Further efforts should be done to enroll new multicentric cohorts to test and validate the herein proposed algorithm.

## Conclusions

Rare malignant laryngeal neoplasms represent a broad spectrum of possible pathologies, each characterized by its particular biological behavior, but generalized laryngeal surgical management principles are still feasible. The TNM staging system seems to efficiently stratify survival, aiding clinicians in critical surgical and medical management decisions. After an adequate diagnostic survey, it is possible to identify patients amenable to conservative surgical treatment and pursue the laryngeal preservation endeavor.

## Supplementary Information

Below is the link to the electronic supplementary material.

Supplementary file1 (PDF 263 kb)

## Data Availability

Full dataset will be available upon reasonable request to the corresponding author.
