# Supplementary material for: Surgical management and oncological outcome of non-squamous cell carcinoma of the larynx: a bicentric study
Source: Eur Arch Otorhinolaryngol. 2021 Sep 24;279(1):299–310. doi: 10.1007/s00405-021-07076-x (PMC8738708; doi:10.1007/s00405-021-07076-x)

SURGICAL MANAGEMENT AND ONCOLOGICAL OUTCOME OF NON-SQUAMOUS CELL CARCINOMA OF THE LARYNX: A MULTICENTRIC STUDY

Supplementary figures

Supplementary Figure S1: Time-to-event outcomes chart showing the follow-up intervals and timeline of event of interest (Total laryngectomy). Circle's size at the time of first surgery is proportional to the overall Stage.

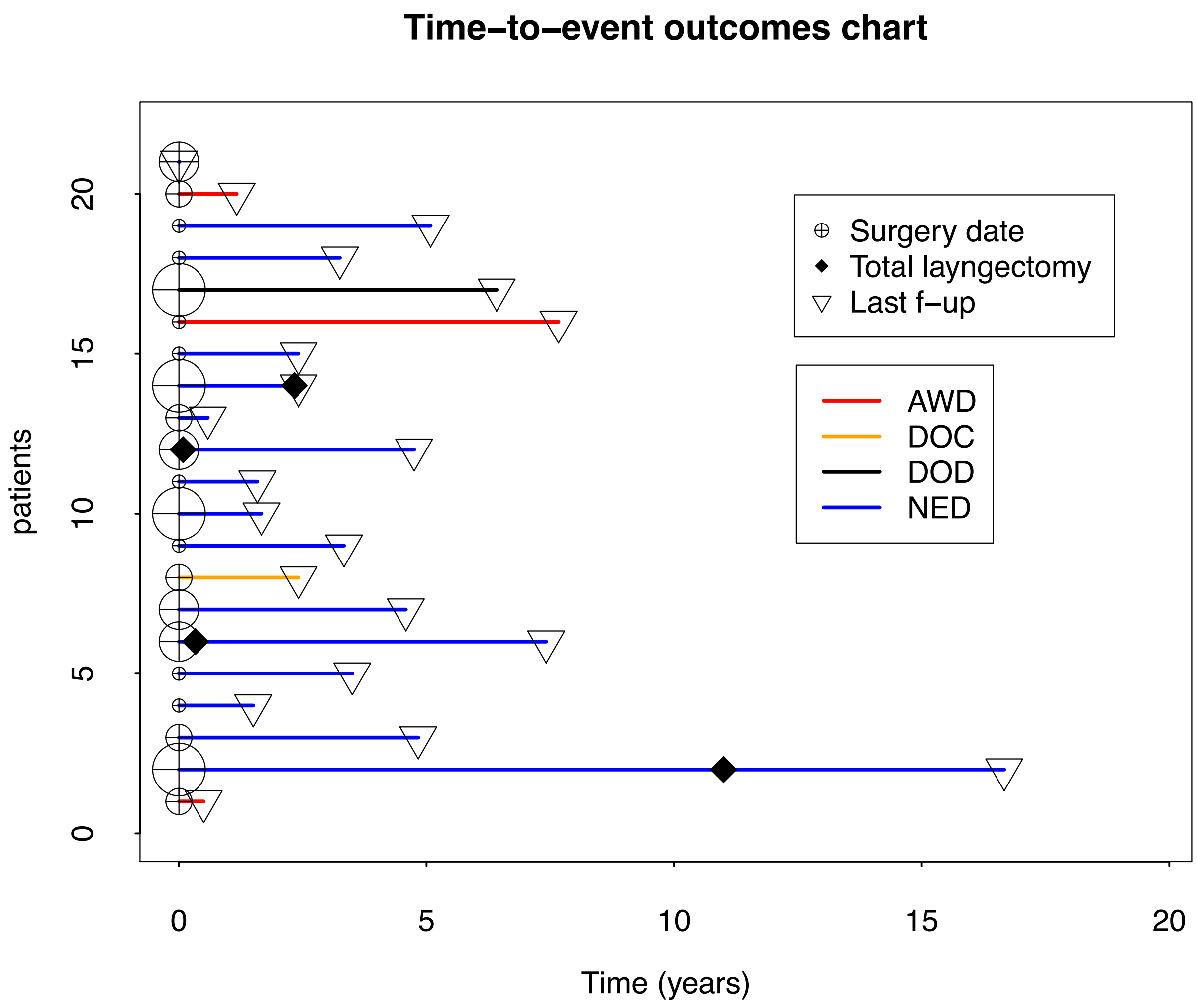

Supplementary Figure S2: Kapan Meier curves of the overall survival considering the type of tumor (**A**), the overall stage (**B**) and the treatment group (**C**). P values estimated by log-rank test. *Legend: SCC, squamous cell carcinoma; TLM, transoral laser microsurgery; OPHL, open partial horizontal laryngectomy; CTRA, crico-tracheal resection and anastomosis; TL, total laryngectomy.*

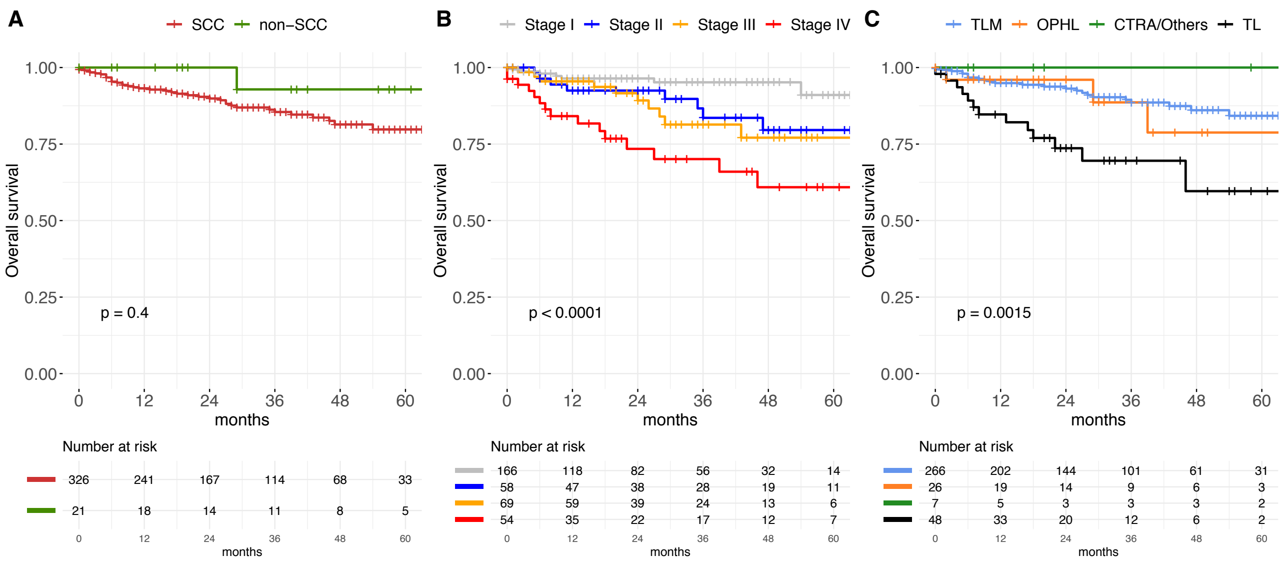

Supplementary Figure S3: Kapan Meier curves of the disease specific survival considering the type of tumor (**A**), the overall stage (**B**) and the treatment group (**C**). P values estimated by log-rank test. *Legend: SCC, squamous cell carcinoma; TLM, transoral laser microsurgery; OPHL, open partial horizontal laryngectomy; CTRA, crico-tracheal resection and anastomosis; TL, total laryngectomy.*

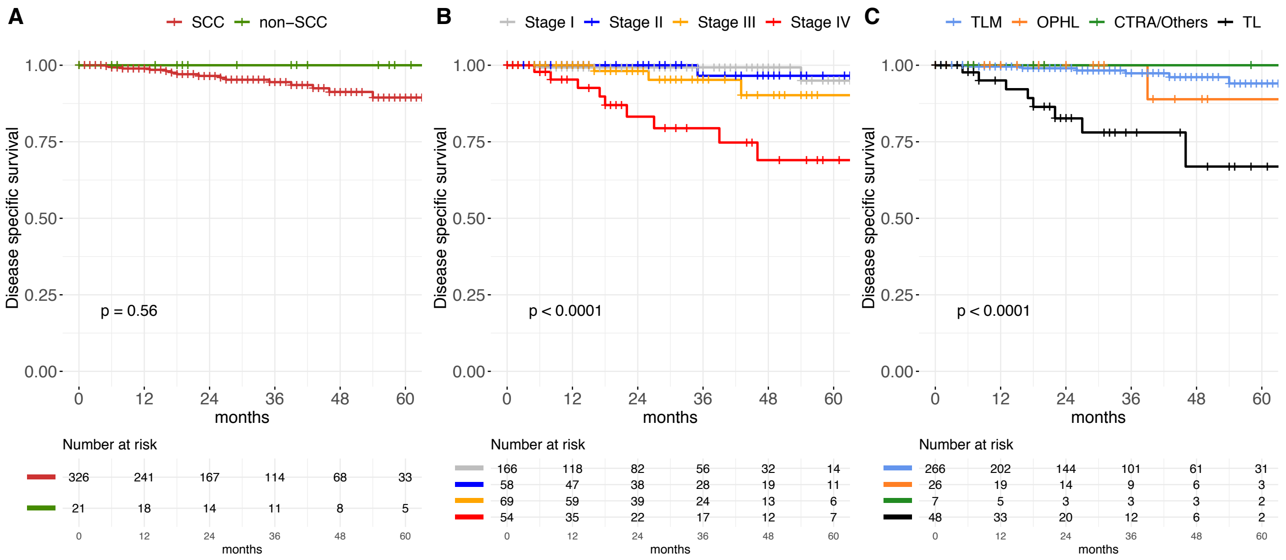

Supplementary Figure S4: Kapan Meier curves of the disease free survival considering the type of tumor (**A**), the overall stage (**B**) and the treatment group (**C**). P values estimated by log-rank test.

Legend: SCC, squamous cell carcinoma; TLM, transoral laser microsurgery; OPHL, open partial horizontal laryngectomy; CTRA, crico-tracheal resection and anastomosis; TL, total laryngectomy.

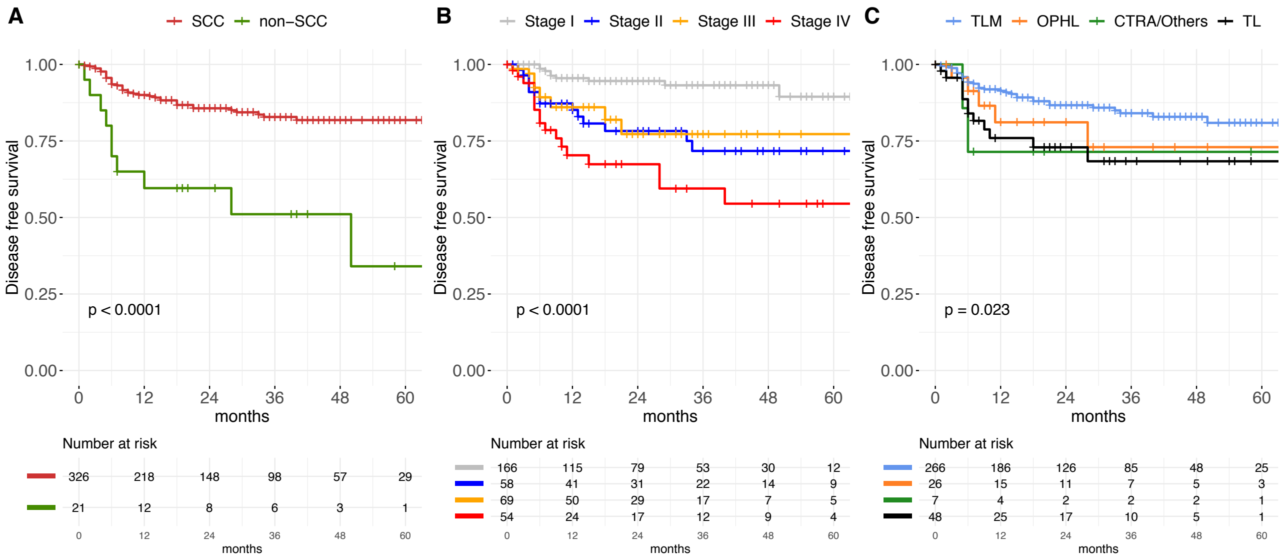

Supplementary Figure S5: Kapan Meier curves of the total laryngectomy free survival considering the type of tumor (**A**), the overall stage (**B**) and the treatment group (**C**). P values estimated by log-rank test.

Legend: SCC, squamous cell carcinoma; TLM, transoral laser microsurgery; OPHL, open partial horizontal laryngectomy; CTRA, crico-tracheal resection and anastomosis; TL, total laryngectomy.

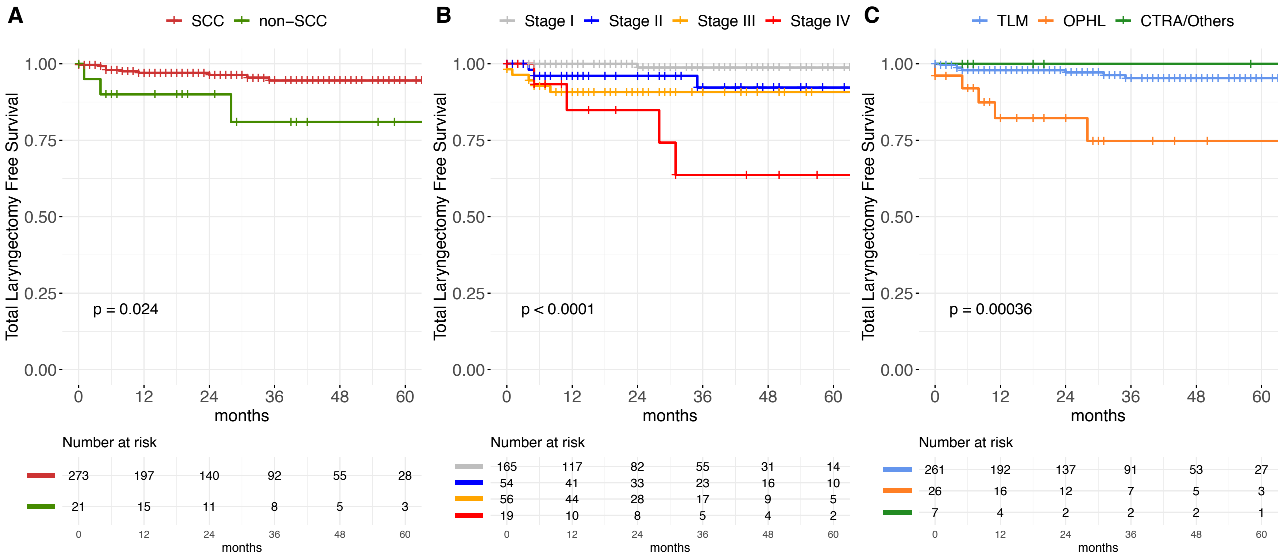

Supplement: Supplementary file 1 — Supplementary file1 (PDF 263 kb) [file 405_2021_7076_MOESM1_ESM.pdf]
